# Supplementary material for: Spread of Jacobaea vulgaris and Occurrence of Pyrrolizidine Alkaloids in Regionally Produced Honeys from Northern Germany: Inter- and Intra-Site Variations and Risk Assessment for Special Consumer Groups
Source: Toxins (Basel). 2020 Jul 7;12(7):441. doi: 10.3390/toxins12070441 (PMC7405020; doi:10.3390/toxins12070441)
Supplement: Supplementary file 1 [file toxins-12-00441-s001.zip › toxins-847763-Table S1.pdf]

Supplementary Materials: Spread of *Jacobaea vulgaris* and Occurrence of Pyrrolizidine Alkaloids in Regionally Produced Honeys from Northern Germany: Inter- and Intra-Site Variations and Risk Assessment for Special Consumer Groups

Christoph Gottschalk, Florian Kaltner, Matthias Zimmermann, Rainer Korten, Oliver Morris, Karin Schwaiger and Manfred Gareis

Table S1. Studies on the occurrence of pyrrolizidine alkaloids (PA) and PA N-oxides (PANO) in honey (raw bulk honey, regionally produced honey and blended retail products) in chronologic order.

Study (year)	Sample type	N	% positive	Origin	Mean PA/PANO level (µg/kg)	Range PA/PANO sum level (µg/kg)	Method	Number of analytes
Deinzer et al. (1977)	bulk honey (*Jacobaea vulgaris*-sourced)	4	100	North America	not reported	300–3900	LC–MS	6
Culvenor et al. (1981)	bulk honey (*Echium plantagineum*-sourced)	5	100	Australia	not reported	270–950	GC–MS	7
Crews et al. (1997)	bulk honey (*Jacobaea vulgaris*-sourced)	15	53	United Kingdom	not reported	<LOD–1480	LC–MS	5
Beales et al. (2004)	bulk honey (*Heliotropium* spp./*Echium* spp.-sourced)	28	100	Australia	not reported	25–2634	LC–MS/MS	17
	bulk honey	35	46			<LOD–814		
	retail honey	5	80			<LOD–1263		
Betteridge et al. (2005)	retail honey	9	78	New Zealand	790	<LOD–2850	LC–MS/MS	15
Kempf et al. (2008)	retail honey	216	8,8	EU/non-EU	56	<LOD–120	GC–MS	retronecine equivalents
Dübecke et al. (2011)	retail honey	696	94	EU/non-EU	26	<LOD–267	LC–MS/MS	10
	bulk honey	2839	68	Central/South America	67	<LOD–1087		

	bulk honey	381	65	EU	26	<LOD–225		
BfR (2011)	retail honey	1324	91	EU/non-EU	21.4	<LOD–267	LC–MS/MS	14/18
	bulk honey	13,280	74	EU/non-EU	55	<LOD–3298		
Kempf et al. (2011)	retail honey	8	75	EU/non-EU	not reported	<LOD–625		
	retail honey	8	100	New Zealand	not reported	334–520	GC–MS	retronecine equivalents
	bulk honey (*Jacobaea vulgaris*-sourced)	31	87	The Netherlands	1261	<LOD–13,019		
Orantes-Bermejo et al. (2013)	regionally produced honey	103	94	Spain	48	<LOD–237	LC–MS/MS	17
Griffin et al. (2013)	retail honey	50	16	EU/non-EU	1260	<LOD–5614	LC–MS/MS	11
Kast et al. (2014)	retail honey	71	54	Switzerland	3.6	<LOD–55	LC–MS/MS	18
Martinello et al. (2014)	retail honey	17	53	Italia	1.4	<LOD–7.0		
		22	50	EU	3.1	<LOD–23	LC–MS	9
		31	77	EU/non EU	17	<LOD–172		
Bodi et al. (2014)	regionally produced honey	15	47	Germany/Austria	6.1	<LOD–28	LC–MS/MS	17
	retail honey	72	93	EU/non-EU	13	<LOD–235		
Griffin et al. (2015 a)	retail honey	59	69	Australia/New Zealand	153	<LOD–932	LC–MS/MS	14
Griffin et al. (2015 b)	retail honey	150	23	EU/non-EU	not reported	< LOD–545	LC–MS/MS	14
Lorena et al. (2016)	bulk honey	60	28	Italy	not reported	<LO–18	LC–MS/MS	6
Valese et al. (2016)	retail honey	92	99	Brazil	not reported	<LOD–423	LC–MS/MS	8
Neumann and Huckauf (2016)	bulk honey	86	54	Schleswig-Holstein	34	<LOD–604	LC–MS/MS	28
Letsyo et al. (2017)	regionally produced honey	48	85	Ghana	283	<LOD–2639	GC–MS	retronecine equivalents
Kowalczyk et al. (2018)	regionally produced honey	40	68	Poland	4.5	<LOD–20	GC–MS	retronecine equivalents
	retail honey	14	93	Asia	23	<LOD–64		
Hungerford et al. (2019)	retail honey	465	84	Australia	280	<LOD–3300	HR–UPLC–MS/MS	30

Carpinelli de Jesus (2019)	regionally produced honey	3	100	Australia	not reported	1400–2000	HR–UPLC–MS/MS	30
Celano et al. (2019)	retail honey	25	100	Italia/EU/non-EU	not reported	0.2–17.5	UPLC–MS/MS	9
