# Supplementary material for: Spread of Jacobaea vulgaris and Occurrence of Pyrrolizidine Alkaloids in Regionally Produced Honeys from Northern Germany: Inter- and Intra-Site Variations and Risk Assessment for Special Consumer Groups
Source: Toxins (Basel). 2020 Jul 7;12(7):441. doi: 10.3390/toxins12070441 (PMC7405020; doi:10.3390/toxins12070441)
Supplement: Supplementary file 1 [file toxins-12-00441-s001.zip › toxins-847763-Table S2 and Table S4.pdf]

Supplementary Materials: Spread of *Jacobaea vulgaris* and Occurrence of Pyrrolizidine Alkaloids in Regionally Produced Honeys from Northern Germany: Inter- and Intra-Site Variations and Risk Assessment for Special Consumer Groups

Christoph Gottschalk, Florian Kaltner, Matthias Zimmermann, Rainer Korten, Oliver Morris, Karin Schwaiger and Manfred Gareis

Table S2. Recovery rates and analytical limits of the LC–MS/MS method for the determination of 25 pyrrolizidine alkaloids (PA) and PA *N*-oxides (PANO) in honey.

Analyte	mean recovery (%) (n=9)	RSD_r (%)	LOD (µg/kg)	LOQ (µg/kg)
Echimidine	102.9	6.7	0.14	0.46
Echimidine *N*-oxide	110.6	16.2	0.02	0.05
Erucifoline	101.8	2.9	0.06	0.19
Erucifoline *N*-oxide	81.8	14.1	0.19	0.59
Europine	92.9	16.1	0.01	0.04
Europine *N*-oxide	102.5	13.0	0.02	0.07
Heliotrine	89.5	11.5	0.08	0.25
Heliotrine *N*-oxide	107.4	3.7	0.01	0.04
Intermedine	88.4	9.2	0.09	0.28
Intermedine *N*-oxide	98.7	7.8	0.10	0.32
Jacobine	94.0	8.8	0.07	0.24
Jacobine *N*-oxide	86.0	13.2	0.01	0.04
Lasiocarpine	98.8	15.5	0.01	0.03
Lasiocarpine *N*-oxide	110.7	17.4	0.02	0.07
Lycopsamine	86.4	6.9	0.02	0.06
Lycopsamine *N*-oxide	100.2	5.0	0.03	0.07
Monocrotaline	96.1	7.1	0.07	0.24
Monocrotaline *N*-oxide	98.4	15.9	0.17	0.54
Retrorsine	106.8	7.8	0.04	0.13
Retrorsine *N*-oxide	120.8	8.0	0.07	0.23
Senecionine	83.0	15.0	0.13	0.40
Senecionine *N*-oxide	106.2	7.8	0.02	0.06
Seneciphylline	111.2	11.5	0.15	0.47
Seneciphylline *N*-oxide	92.8	22.6	0.02	0.05
Senkirkine	102.4	5.1	0.01	0.04

RSDr intra-day repeatability expressed as relative standard deviation; LOD, limit of detection; LOQ, limit of quantification.

Table S4. Substance specific parameters of the LC–MS/MS method for 25 pyrrolizidine alkaloids (PA) and PA *N*-oxides (PANO) ordered by retention time.

Analyte	Formula	Precursor ion (m/z)	Product ions (m/z)	DP (V)	EP (V)	CE (eV)	CXP (V)	Retention time (min)
Mc	$C_{16}H_{23}NO_6$	326.1	121.1	116	10	41	20	5.2
			120.2	116	10	49	20	
Im	$C_{15}H_{25}NO_5$	300.2	138.0	96	10	29	24	6.4
			120.1	96	10	37	20	
Ly	$C_{15}H_{25}NO_5$	300.2	138.0	96	10	29	24	6.9
			120.1	96	10	37	20	
Eu	$C_{16}H_{27}NO_6$	330.1	138.0	86	10	41	6	8.8
			156.1	86	10	41	8	
McN	$C_{16}H_{23}NO_7$	342.1	137.1	116	10	41	24	9.9
			120.1	116	10	51	20	
ImN	$C_{15}H_{25}NO_6$	316.2	172.1	61	10	41	30	9.9
			138.1	61	10	41	24	
EuN	$C_{16}H_{27}NO_7$	346.2	172.1	106	10	45	30	9.8
			270.1	106	10	35	16	
LyN	$C_{15}H_{25}NO_6$	316.2	172.1	106	10	41	30	10.0
			138.0	106	10	41	24	
Ec	$C_{18}H_{23}NO_6$	350.2	120.1	111	10	43	20	10.7
			138.1	111	10	49	20	
Jb	$C_{18}H_{25}NO_6$	352.1	120.1	111	10	43	20	11.2
			280.2	111	10	33	16	
Ht	$C_{16}H_{27}NO_5$	314.2	138.2	86	10	41	36	12.7
			94.0	86	10	41	16	
JbN	$C_{18}H_{25}NO_7$	368.2	296.2	111	10	35	16	12.7
			120.1	111	10	53	20	
EcN	$C_{18}H_{23}NO_7$	366.2	118.0	111	10	49	20	12.7
			120.1	111	10	45	22	
Rs	$C_{18}H_{25}NO_6$	352.1	138.1	121	10	43	24	12.9
			324.3	121	10	39	18	
HtN	$C_{16}H_{27}NO_6$	330.2	172.1	106	10	41	30	13.6
			93.7	106	10	63	16	
Sp	$C_{18}H_{23}NO_5$	334.1	120.1	111	10	41	20	14.1
			94.1	111	10	51	16	
RsN	$C_{18}H_{25}NO_7$	368.2	118.1	111	10	47	20	14.1
			136.0	111	10	51	22	
Sc	$C_{18}H_{25}NO_5$	336.2	120.1	116	10	41	20	16.9
			138.1	116	10	41	24	
SpN	$C_{18}H_{23}NO_6$	350.2	118.2	116	10	51	20	16.9
			136.0	116	10	45	24	
Em	$C_{20}H_{31}NO_7$	398.3	120.1	96	10	35	20	18.1
			119.5	96	10	37	42	
ScN	$C_{18}H_{25}NO_6$	352.1	118.1	116	10	41	20	18.3
			136.0	116	10	53	24	
Sk	$C_{19}H_{27}NO_6$	366.2	122.1	121	10	49	20	18.4
			153.0	121	10	37	26	
EmN	$C_{20}H_{31}NO_8$	414.2	254.2	106	10	43	14	18.4
			352.2	106	10	35	20	
Lc	$C_{21}H_{33}NO_7$	412.2	120.1	101	10	45	22	19.5
			220.1	101	10	27	12	
LcN	$C_{21}H_{33}NO_8$	428.2	137.1	111	10	47	24	20.1
			138.1	111	10	45	24	

Ec, erucifoline; EcN, erucifoline *N*-oxide; Em, echimidine; EmN, echimidine *N*-oxide; Eu, europine; EuN, europine *N*-oxide; Ht, heliotrine; HtN, heliotrine *N*-oxide; Im, intermedine; ImN, intermedine *N*-oxide; Jb, jacobine; JbN, jacobine *N*-oxide; Lc, lasiocarpine; LcN, lasiocarpine *N*-oxide; Ly, lycopsamine; LyN, lycopsamine *N*-oxide Mc, monocrotaline; McN, monocrotaline *N*-oxide; Rs, retrorsine; RsN, retrorsine *N*-oxide; Sc, senecionine; ScN, senecionine *N*-oxide; Sp, seneciphylline; SpN, seneciphylline *N*-oxide; Sk, senkirkine.
